# β-mercaptoethanol assists efficient construction of sperm bacterial artificial chromosome library

**DOI:** 10.14440/jbm.2017.167

**Published:** 2017-01-20

**Authors:** Kohei Fujikura, Masanori Abe, Reiko Kuroda

**Affiliations:** ^1^Department of Life Sciences, Graduate School of Arts and Sciences, The University of Tokyo, Tokyo, Japan; ^1^JST ERATO-SORST Kuroda Chiromorphology Project, The University of Tokyo, Tokyo, Japan; ^1^Department of Biophysics and Biochemistry, Graduate School of Science, The University of Tokyo, Tokyo, Japan; ^1^Research Institute for Science and Technology, Tokyo University of Science, Tokyo, Japan

**Keywords:** BAC library, DNA extraction, large-insert, sperm, β-mercaptoethanol

## Abstract

Bacterial artificial chromosome (BAC) library plays a critical role in the strategic research in genomics. Sperm is known as a good source for BAC library construction. However, preparation of intact DNA from the highly condensed sperm nuclei is not easy. Here we developed and validated an efficient DNA extraction strategy for BAC library construction from sperm embedded in agarose plugs. The protocol used a combination of lauroylsarcosine, proteinase K and β-mercaptoethanol (a reducing agent of nucleus) In comparison with the normal protocol without reducing agents, β-mercaptoethanol released high-molecular-weight DNA from the protamines which permit DNA to be packed very densely within the spermatozoan nucleus, without damaging DNA. Extracted DNA by this method was readily digested by restriction enzymes and ideal for BAC library construction.

## INTRODUCTION

Large-insert Bacterial artificial chromosome (BAC) libraries are necessary for the advance in genetic and genomic research, particularly for higher eukaryotes [1-6]. The BAC library has opened the way for genome analysis and systematic studies of gene loci. However, the process of BAC library construction remains relatively challenging and time consuming despite improvements in the methodology [7-11]. One of the most important steps is the isolation of high-molecular-weight DNA (HMW-DNA). Whereas some HMW-DNA preparation methods have been shown to be applicable to some specific cases [7-11], there are not many reports which clearly give us the biotechnical information about BAC library construction. Thus it is often challenging to extract HMW-DNA condensed in sperm nuclei by simply applying the techniques generally used for somatic cells.

Sperm is known as a good source for BAC library construction because it does not contain inhibitors of reactions including ligation. Furthermore mitochondrial and bacterial DNA contaminations are speculated to be minimal (Each sperm carries only four mitochondria). However, it is still difficult to prepare the HMW-DNA from the highly condensed nuclei spermatozoa, especially in the case of animals with internal fertilization. Difficulties arise from the presence of the small proteins called protamines which are the basic proteins and resistant to chemical disruption [12-14]. During spermatogenesis, spermatocyte histones are replaced by protamines and DNA is highly condensed in the spermatozoan nucleus. When the protamines are not destroyed, DNA within sperm nucleus is not readily accessible by any DNA restriction enzyme. In this study, we have validated a rapid and efficient method using β-mercaptoethanol for extracting HMW-DNA from sperms embedded in agarose plugs.

## MATERIALS AND METHODS

All procedures in this study were approved by the Experimental Guidelines of The University of Tokyo. Sperms were freshly prepared from animal testis (in this case freshwater gastropod *Lymnaea stagnalis*). They were washed in phosphate-buffered saline (PBS: 0.8% NaCl, 0.02% KCl, 0.144% Na_2_HPO_4_, 0.024% KH_2_PO_4_) twice, harvested by centrifugation, and then resuspended in PBS to a final sperm density of 1.0 × 10^8^ cells/ml. The sperm suspension was then mixed with an equal volume of 1% InCert agarose (FMC Bioproducts, USA) in PBS and poured into a disposable plug mold (Bio-Rad, USA), resulting in 10 × 5 × 1.5 mm agarose plugs, as described previously [7,8].

Agarose plugs were then treated with a modified buffer system including 2% N-lauroyl sarcosine, 0.4 M EDTA (pH 8.0), 2 mg/ml Proteinase K (TAKARA, Japan), and 0.2% β-mercaptoethanol at 55^°^C for 12 h. Discolored plugs were then transferred to a new dish and treated with 2% N-lauroyl sarcosine, 0.4 M EDTA (pH 8.0), and 2 mg/ml Proteinase K at 55^°^C for 12 h to remove the β-mercaptoethanol. After the proteinase K digestion, agarose plugs were washed in TE [10 mM Tris, 1 mM EDTA (pH 8.0)] about 5 times.

Then restriction digestion was performed with Hind III endonuclease (New England Biolabs, USA) at 37^°^C for 20 min. Partially digested agarose plugs were run in a 1% agarose gel (Sigma Type II; Sigma, USA) using a CHEF apparatus (BioRad, USA), with 0.5 × TBE buffer. All DNA samples were separated on a 0.5 × TBE 1% agarose gel. PFGE conditions were as follows: voltage = 6 V/cm, switch time = 5 s to 50 s, run time = 16 h, T = 14^°^C, angle = 120^°^. After electrophoresis, the gel was stained in 0.5 mg/ml ethidium bromide solution. Approximately 150 to 250 kb of HMW-DNA in agarose was isolated by cutting, and this agarose gel was then melted by adding one-half weight of sodium iodide and leaving it overnight. To remove sodium iodide and agarose from this DNA solution, float dialysis was performed using a filter membrane (pore size, 0.025 μm; Millipore, USA), which was floating on prewarmed TE buffer in a petri dish at 45^°^C d for 15 min. One unit of GELase (Epicentre, USA) was added to the DNA solution and left for 15 min. The DNA solution was transferred to which 2 U of additional GELase was added and kept for 2 h at 45^°^C. The concentration and state of purified HMW-DNA was estimated by using 0.8% agarose gel electrophoresis.

Extracted HMW-DNA was ligated to 5 ng of dephosphorylated pBAC-lac at a 1:10 molar ratio of insert to vector with 0.1 U of T4 DNA ligase (Thermo Fisher Scientific, USA) in a 25 μl total volume. For ligation conditions, 10 cycles of 30 min at 22^°^C, 30 min at 14^°^C, and 30 min at 4^°^C were performed. The ligation mixture was spotted onto the dialysis filter floating on 0.2 × TE buffer in a petri dish for 1 h at room temperature. 10 μl of dialyzed ligation mixture was used to transform 35 μl of DH10B competent cells by electroporation. Transformation was performed using Electro Cell Manipulator (BTX, USA) under the following conditions: (voltage booster settings) resistance on voltage booster, 129 ohms; (cell porator settings) voltage gradient, 14 kV/cm; and capacitance, 50 micro F in the high voltage condition. Cells were incubated by shaking for 40 min at 37^°^C in 1 ml of SOC medium. They were then plated on 500 cm^2^ LB agar plates containing chloramphenicol (12.5 μg/ml), X-gal (40 μg/ml), and IPTG (100 μg/ml). The plates were incubated at 37^°^C overnight.

Blue and white color selection was used to identify the recombinant clones. White BAC colonies were automatically picked in 128 microtiter plates with 384 wells (approximately 100000 clones) using Q-Pix (Molecular devices, USA) containing 80 μl of LB with 12.5 μg/ml chloramphenicol in each well. The plastic plates were incubated at 37^°^C overnight. Each 384-well microtiter plate was replicated 3 times using a BioGrid (Biosurplus, USA) and stored at −80^°^C.

On hundred BACs were randomly selected and redigested for insert size assessment by pulsed-field electrophoresis. BAC DNA was isolated using a QIAwell Midi prep Plasmid Kit (Qiagen, USA) following the protocol of the manufacturer. Isolated BAC clones were digested with the restriction enzyme Not I (New England Biolabs) and then subjected to PFGE for 14 h using the same PFGE conditions as shown above.

## RESULTS AND DISCUSSION

**Figure 1.**
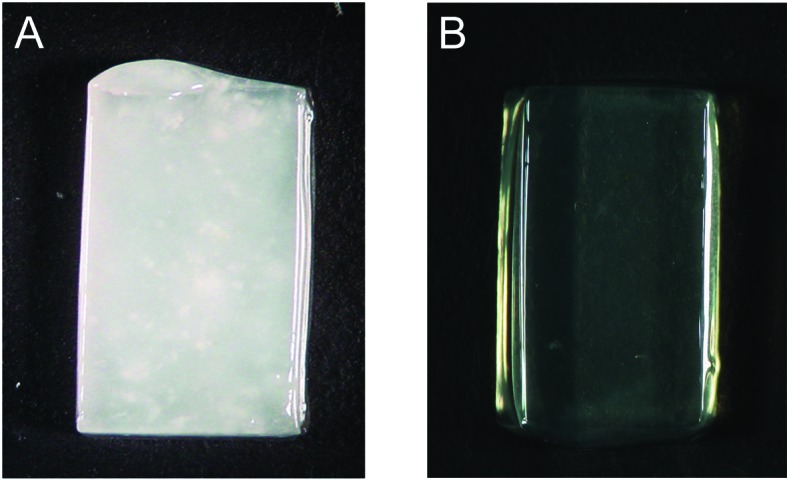
**Agarose plugs after proteinase K and lauroylsarcosine treatment without or with β-mercaptoethanol.** Sperm nuclei embedded in agarose plugs were treated without (**A**) or with (**B**) β-mercaptoethanol, and the colors of the plugs were compared. White plugs containing condensed nuclei spermatozoa become clear after β-mercaptoethanol treatment.

Agarose plugs containing sperms were white due to the highly condensed nuclei spermatozoa. The plugs treated with the medium containing β-mercaptoethanol then became clear and could be used for downstream applications (**Fig. 1**). This is caused by swelling of nuclei spermatozoa, which was initially observed during the first 1 h of incubation. In contrast agarose plugs treated only with proteinase K and lauroylsarcosine for up to 24 h were still unclear (**Fig. 1**), thus suggesting that it is difficult to release the DNA from the nuclei spermatozoa without disulfide bond reducing agent.

**Figure 2.**
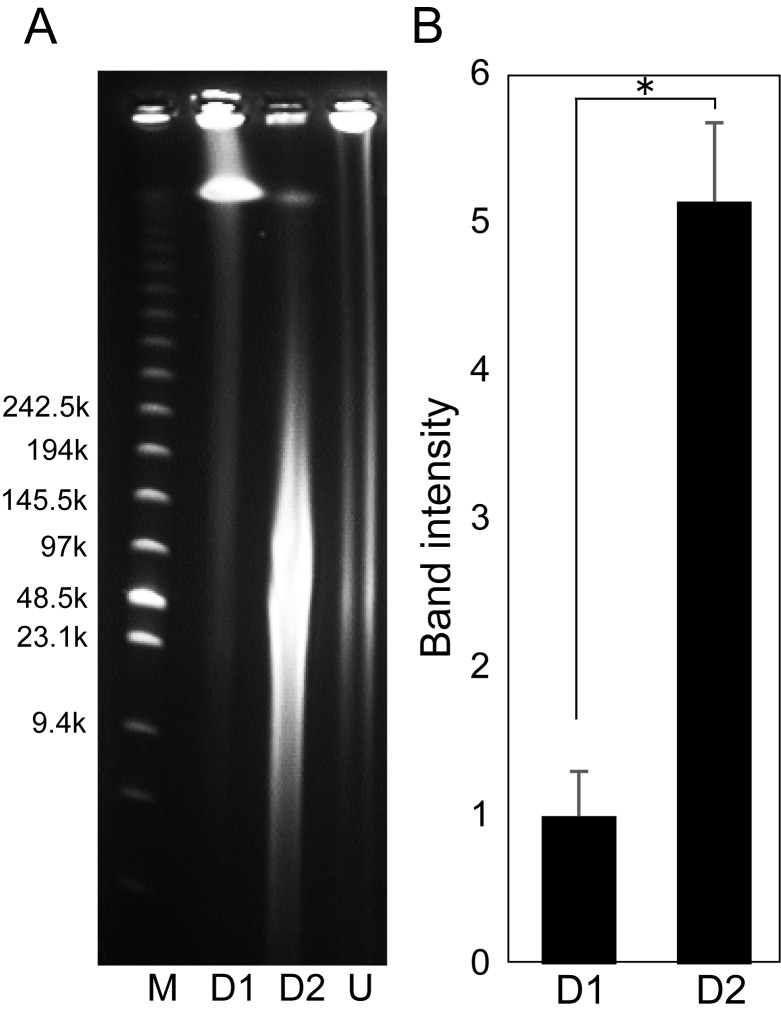
**Pulse-field gel electrophoresis analysis of partially digested sperm DNA treated without or with β-mercaptoethanol. A.** Agarose plugs treated in the absence (Lane D1) or presence (Lane D2) of β-mercaptoethanol were digested with 6 units of Hind III at 37^°^C for 20 min. β-mercaptoethanol treatment increased the amount of accessible DNA. Undigested plugs were electrophoresed in Lane U. The low range PFG marker (Lane M) was run alongside with three lanes. **B.** Relative band intensity of Hind III digested DNA. Band intensity in the gel electrophoresis was measured in the range of 100–500 kbp and compared between the absence (Lane D1) and presence (Lane D2) of β-mercaptoethanol. *n* = 3, *P < 0.05.

HMW-DNA treated with β-mercaptoethanol was easily subjected to restriction enzyme digestion (**Fig. 2**). Furthermore few degradation products were observed. On the other hands, β-mercaptoethanol non-treated plugs prove to be recalcitrant to restriction enzyme (**Fig. 2**). β-mercaptoethanol irreversibly denatures the highly condensed protamines by reducing disulfide bonds and destroying the native conformation required for protein functionality. When the protamines are destroyed, DNA within sperm nucleus becomes readily accessible. The important point is that β-mercaptoethanol did not influence DNA purity and length. Only a few reports have described the use of β-mercaptoethanol for BAC library construction [15-18], and thus β-mercaptoethanol is uncommon chemical regent for large-insert library construction. The reason for use has not been described at all, and the usage, efficiency and side effects of β-mercaptoethanol have never been assessed in detail. Here we showed the significance of β-mercaptoethanol in the BAC library construction and, as far as we know, this is the first application of β-mercaptoethanol to sperm BAC library.

**Figure 3.**
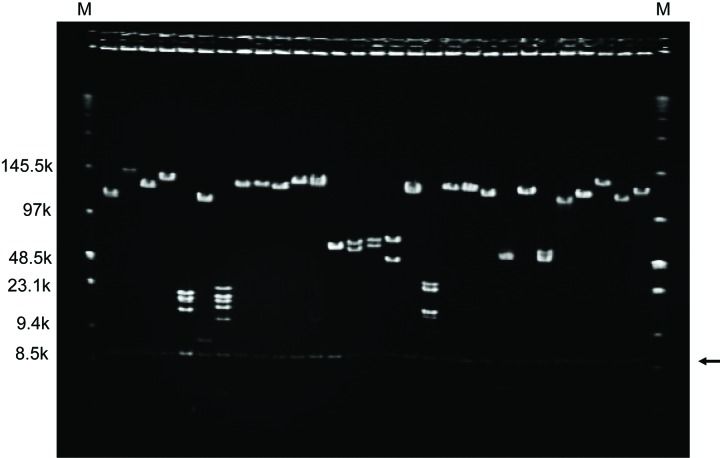
**Insert size assessment of BAC clones by pulsed-field gel electrophoresis.** Twenty-nine BAC clones were randomly selected, digested using Not I enzyme and electrophoresed using PFGE system (Lane 1–29). pBAC-lac vector was positioned at the 5.6 kb (arrow). The low range PFG marker (Lane M) was run alongside with twenty nine BAC clones.

This method was actually applied to the construction of *L. stagnalis* sperm BAC library, which comprises over 100000 clones with an average insert size of 135 kb without contamination of mitochondrial and bacterial DNA (**Fig. 3**) [18]. We have successfully constructed the 700 kbp contig of target region and identified the handedness-determining gene of *L. stagnalis* [18]. We also confirmed that β-mercaptoethanol completely degrades mouse sperm nucleus in agarose plugs (**Fig. S1**; the same protocol was carried out), illustrating the versatility of this technique. Further research is necessary to confirm the broad availability of this technique.

In conclusion, β-mercaptoethanol is necessary for preparing sperm HMW-DNA, suitable for BAC library construction from metazoa, particularly non-model organisms with no available cell lines. The method presented here utilizes a combination of β-mercaptoethanol, lauroylsarcosine and proteinase K to degrade the sperm nuclei extract, after embedding it in agarose plugs. The isolated HMW-DNA is of high quality, accessible to restriction enzyme digestions, and allows cloning of DNA fragments with an average size of 100 kb or more.

## Abbreviations used

BAC: bacterial artificial chromosome
HMW-DNA: high-molecular-weight DNA

## Supplementary information

**Figure S1**. Agarose plugs from mouse sperm were treated with or without β-mercaptoethanol.
